# Intracellular and extracellular S100A9 trigger epithelial-mesenchymal transition and promote the invasive phenotype of pituitary adenoma through activation of AKT1

**DOI:** 10.18632/aging.104072

**Published:** 2020-11-17

**Authors:** Ning Huang, Guanjian Zhao, Qiang Yang, Jiahe Tan, Ying Tan, Jiqin Zhang, Yuan Cheng, Jin Chen

**Affiliations:** 1 Department of Neurosurgery, The Second Affiliated Hospital of Chongqing Medical University, Chongqing, China; 2 Department of Neurosurgery, Guizhou Provincial People’s Hospital, Guiyang, Guizhou, China; 3 Department of Anesthesiology, Guizhou Provincial People's Hospital, Guiyang, Guizhou, China

**Keywords:** pituitary adenoma, S100A9, AKT1, epithelial-mesenchymal transition, invasion

## Abstract

Pituitary adenoma (PA) is mostly benign intracranial tumor, but it also displays invasive growth characteristics and provokes challenging clinical conditions. S100A9 protein enhances tumor progression. In this study, we firstly demonstrated that both intracellular and extracellular S100A9 promoted the expression of Vimentin and Intercellular cell adhesion molecule-1 (ICAM-1), coupled with reduced E-cadherin in PA. As a result, PA acquired the phenotype of Epithelial-Mesenchymal Transition (EMT), leading to proliferation, cell cycle progression, migration and invasion. In addition, we indicated S100A9-induced EMT was mediated by activation of AKT1. Furthermore, immunohistochemistry showed that S100A9 expression was higher in invasive PA than that in non-invasive PA. These data extended our understanding for the effects of S100A9 on PA invasion and contributed to further development of a promising therapeutic target for invasive PA.

## INTRODUCTION

Pituitary adenoma (PA) is a common neuroendocrine neoplasm and consists of 10-15% of primary tumors in central nervous system [1]. In spite of having benign nature, one third of PA manifests aggressive behaviors, resulting in infiltration of the cavernous sinus, sphenoid and dura [2]. Such tumors are defined as invasive pituitary adenoma (IPA). Owing to the large size of these tumors and serious destruction of adjacent structures, it is difficult to remove IPA completely, giving rise to the presence of residual tumor, high recurrence and poor prognosis [3]. Accordingly, it is indispensable to expand our understanding of PA molecular pathogenesis to develop novel targets for therapy.

S100A9, a member of the S100 family of EF-hand motif Ca^2+^-binding proteins, is located near a break-point region on chromosome 1q21 where the gene deletion, translocation and overlap have been frequently found to link with malignancy [4]. Upregulation of S100A9 has been observed in some tumors, such as glioma, hypopharyngeal cancer, lung cancer, breast carcinoma, hepatocellular carcinoma, gastric cancer, pancreatic tumor, colorectal cancer, bladder cancer, prostate cancer, ovarian cancer, cervical cancer and osteosarcoma [5–11]. Overexpression of S100A9 heightened tumorigenesis, tumor progression and recurrence [12–14]. It is universally acknowledged that S100A9 has both intracellular and extracellular functions in tumor microenvironment. Many studies have shown that intracellular S100A9 activates several tumor-associated signaling pathways and upgrades the proliferation of tumor cells [6, 11]. Furthermore, both tumor cells and immune cells are able to secrete the S100A9 protein, stimulating the recruitment of myeloid-derived suppressor cells (MDSC) and the formation of a pre-metastatic niche in the tumor stroma [15]. The extracellular S100A9 protein also acts as a ligand and interacts with surface receptors on tumor cells, including the receptor for advanced glycation end product (RAGE), Toll-like receptor 4 (TLR4) and CD147. These bindings accelerate tumor proliferation and infiltration [16]. Our earlier data also revealed that inhibition of S100A9 represented a promising strategy for the treatment with neoplasm [5, 11, 17].

AKT1 plays a vital role in S100A9 downstream signal transduction pathway in favor of tumor progression, including PA [18–20]. Increasing evidence has demonstrated that constitutive activation of AKT1 induced epithelial-mesenchymal transition (EMT), which is an essential step in tumor invasion [21]. During the EMT process, tumor cells were endowed with enhanced motility and attenuated intercellular adhesion in order to migrate and diffuse to surrounding structures [22]. Interestingly, a few reports have indicated that EMT was associated with invasive characteristics of PA [18], but the elements related to EMT trigger are not well understood.

In this study, we showed that PA invasion was amplified by increased levels of intracellular and extracellular S100A9. Furthermore, we confirmed that S100A9 activated AKT1 and promoted EMT transition. These findings support that targeting S100A9 will be conducive to novel strategy for IPA molecular therapy toward residual and recurring tumors after surgical resection.

## RESULTS

### Extracellular S100A9 activates AKT1 and facilitates EMT in HP75 cells

**Figure 1:**
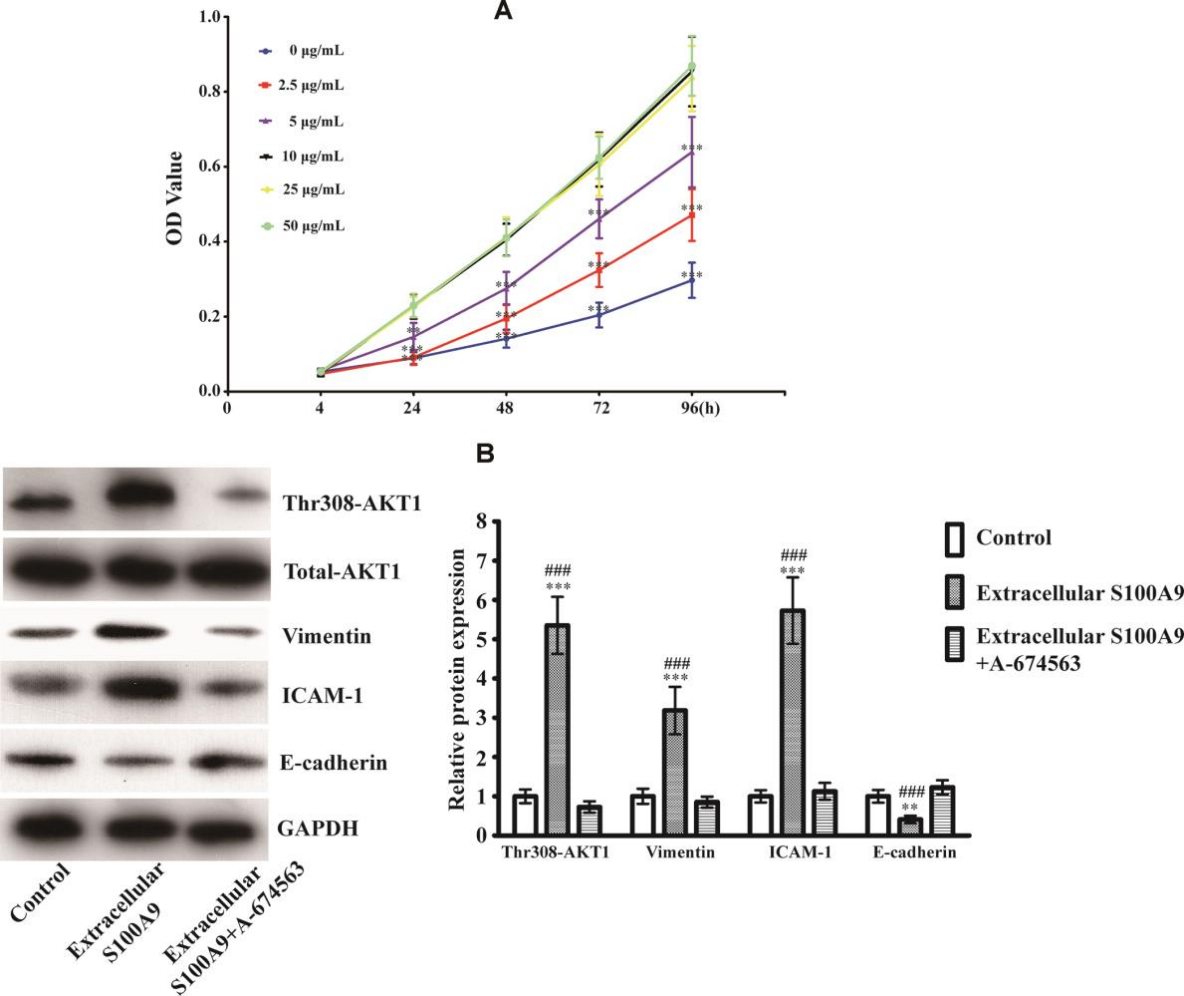
**Expression of EMT-related proteins after treatment with the recombinant human S100A9 protein and AKT1 inhibitor.** (**A**) After incubation with different concentrations of recombinant human S100A9 protein for 24, 48, 72 and 96h, the viability of HP75 cells was assessed using CCK-8(n=5. *P*<0.01^**^; *P*<0.001^***^, compared with the group with 10μg/mL recombinant S100A9 protein at the same time point). (**B**) HP75 cells were divided into control group, 10μg/mL recombinant S100A9 protein group and 10μg/mL recombinant S100A9 protein+ A-674563 group, the levels of p-AKT1^Thr308^, Vimentin, ICAM-1 and E-cadherin were observed using western blot(n=5. *P*<0.01^**^; *P*<0.001^***^ versus control group. *P*<0.001^###^ versus 10μg/mL recombinant S100A9 protein+ A-674563 group).

Previous studies have unveiled that 10μg/mL recombinant human S100A9 protein promoted tumor development [23–25]. We also found that the cell viability of HP75 was induced in a S100A9 concentration-dependent manner (0, 2.5, 5, 10μg/mL). However, extracellular S100A9 did not further accelerate HP75 proliferation upon increasing its concentration from 10 to 50μg/mL (Figure 1A). Accordingly, 10μg/mL recombinant human S100A9 protein was used for subsequent experiments. Western blot analysis demonstrated that extracellular S100A9 raised phospho-AKT1^Thr308^, Vimentin and ICAM-1 (Intercellular cell adhesion molecule-1), coupled with downregulation of E-cadherin. When A-674563 (AKT1 inhibitor) was added, elevated Vimentin and ICAM-1 were suppressed partially. Conversely, the levels of E-cadherin were increased under these conditions (Figure 1B). These data suggest that extracellular S100A9 triggers off EMT of PA in an AKT1^Thr308^-dependent manner.

### Extracellular S100A9 causes proliferation, migration and invasion of HP75

**Figure 2:**
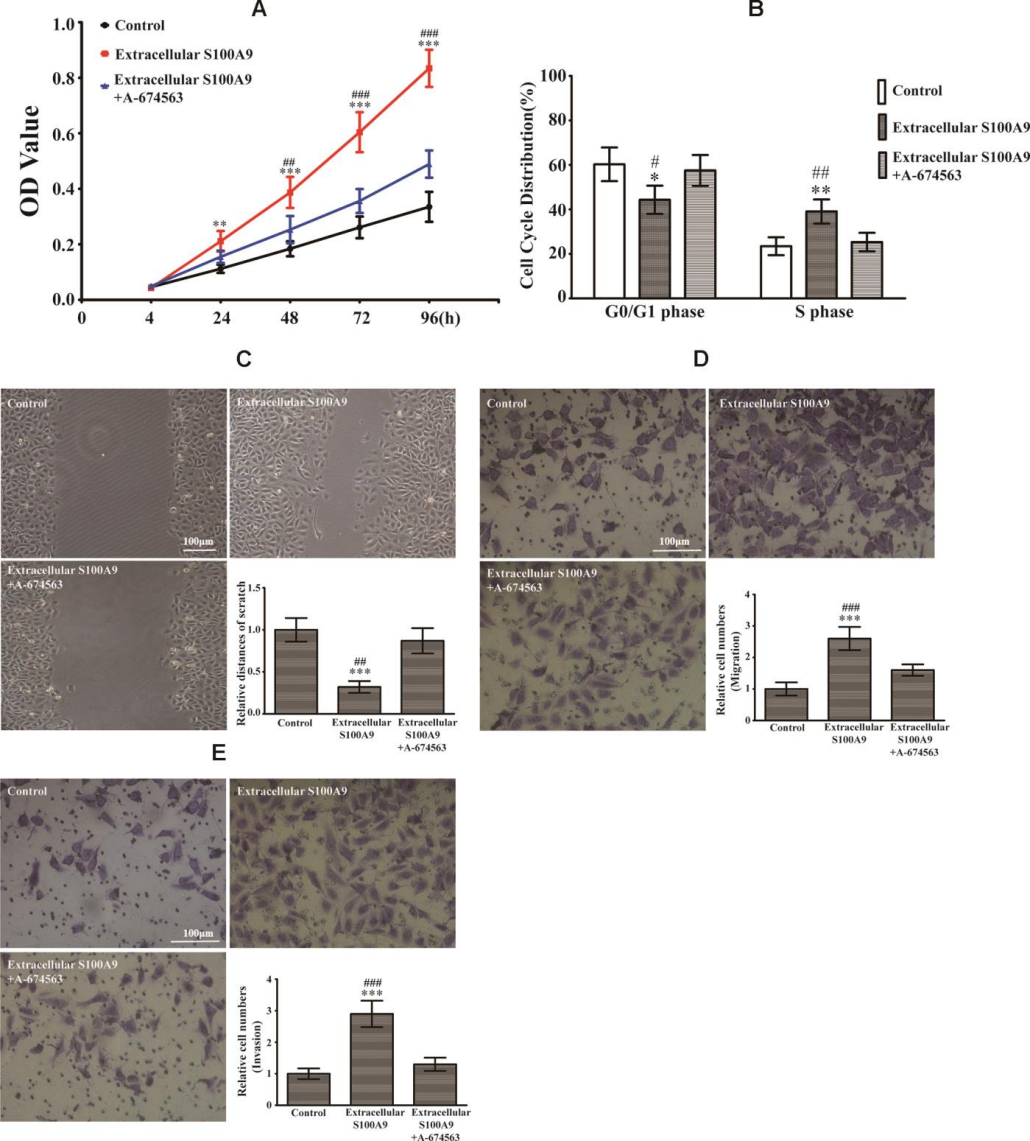
**Extracellular S100A9 participated in proliferation, migration and invasion of PA through activation of AKT1.** (**A**) The HP75 cells were divided into control group, recombinant S100A9 protein group and recombinant S100A9 protein+ A-674563 group. CCK-8 showed that the recombinant S100A9 protein accelerated proliferation at 24, 48, 72 and 96h. However, the recombinant S100A9 protein-mediated promotion of proliferation was partially reversed by the addition of an AKT1 inhibitor(A-674563). (**B**) Flow cytometry indicated that the recombinant S100A9 protein decreased the G0/G1 phase proportion and increased the S phase ratio, but these effects could be counteracted by A-674563. (**C**, **D**) The cell migration was analyzed by a wound-healing assay and a transwell insert system after treatment with the recombinant S100A9 protein alone or combined with A-674563. (**E**) The transwell assay also demonstrated that the recombinant S100A9 protein improved invasion of HP75 cells, but the function could be neutralized by A-674563. (n=5. ^*^P<0.05; ^**^P<0.01; ^***^P<0.001 *vs*. Control group. ^#^*P*<0.05; ^##^*P*<0.01; ^###^*P*<0.001 *vs*. Recombinant S100A9 protein+ A-674563 group).

The CCK-8 results illuminated that extracellular S100A9 promoted HP75 proliferation after 24, 48, 72 and 96h (Figure 2A). Flow cytometric analysis indicated that the percentage of cells in the G0/G1 phase was lower in HP75 cells treated with extracellular S100A9 than in the control group, while the percentage of cells that in the S phase was higher (Figure 2B). However, there was no significance difference in the percentage of cells that were in the G2 phase (data not shown). Moreover, the transwell and wound-healing assay also illustrated that extracellular S100A9 heightened migration and invasion of HP75 cells after 16h (Figure 2C–2E). Interestingly, extracellular S100A9-induced proliferation, cell cycle progression, migration and invasion were counteracted by the addition of A-674563 in HP75 cells (Figure 2A–2E).

### Intracellular S100A9 leads to EMT via phosphorylation of AKT1^Thr308^ in HP75

**Figure 3:**
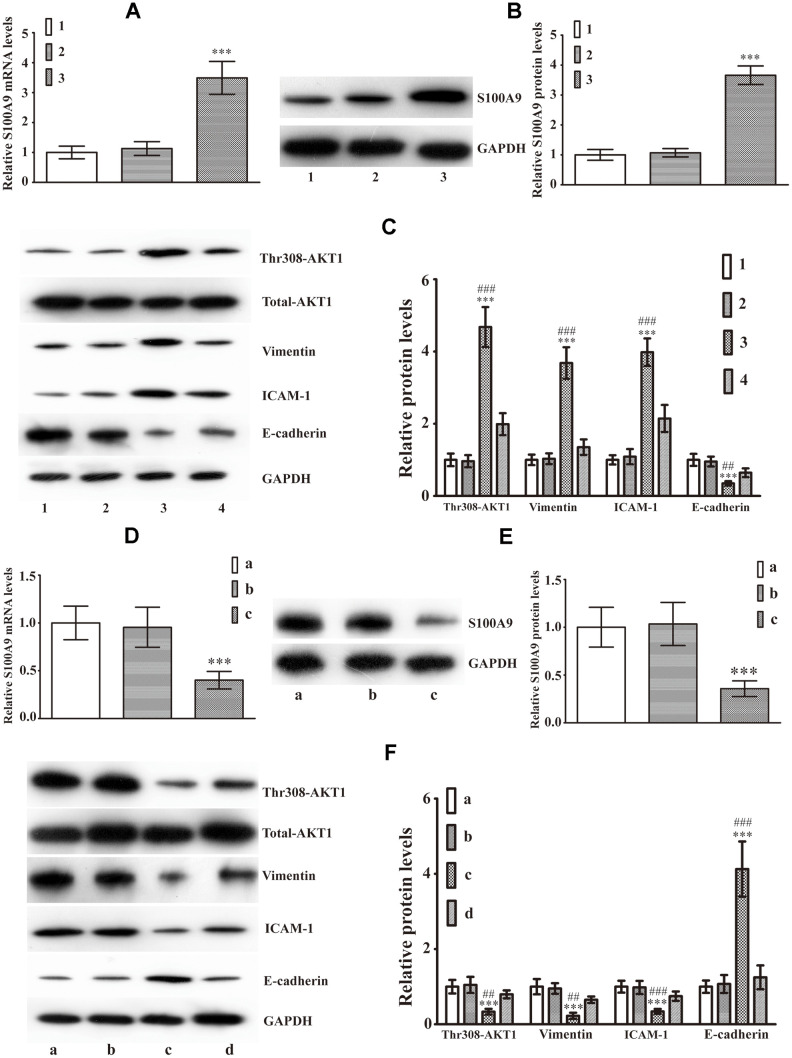
**Intracellular S100A9 regulates phosphorylation of AKT1^Thr308^ and induces EMT in PA**. (**A**) The mRNA levels of S100A9 in control group (group 1), NC1 group (group 2), LV-S100A9 group (group 3) were detected by Real-Time PCR(n=9, P^***^<0.001 versus group 1). (**B**) Western blot was used to observe S100A9 protein levels in groups 1, 2, and 3(n=5, P^***^<0.001 versus group 1). (**C**) The expression of phospho-AKT1^Thr308^, Vimentin, ICAM-1 and E-cadherin was explored using western blot analysis in groups 1, 2, 3 and a LV-S100A9 + addition of A-674563 group (group 4)(n=5, P^***^<0.001 *vs*. group 1. *P*^##^<0.01; *P*^###^<0.001 *vs*. group 4). (**D**, **E**) Real-time PCR and western blot analysis were used to investigate S100A9 mRNA and protein expression in the control group (group a), NC2 group (group b) and S100A9-shRNA-LV group (group c), respectively(n=5, P^***^<0.001, compared with group a). (**F**) Western blot demonstrated that downregulation of S100A9 reduced phospho-AKT1^Thr308^, Vimentin, ICAM-1, coupled with increase in E-cadherin. The S100A9-shRNA-LV + SC79(AKT1 agonist) group (group d) displayed that the reduced phospho-AKT1^Thr308^, Vimentin, ICAM-1 and elevated E-cadherin were reversed partially by SC97(n=5. P***<0.001, compared with group a. P##<0.01; P###<0.001, compared with group d).

We extended the study to analyze the effects of intracellular S100A9 on EMT in HP75 cells. Real-time PCR and western blot analysis verified that both mRNA and protein levels of S100A9 were markedly elevated in cells transfected with LV-S100A9 compared with cells transfected with a negative control lentivirus vector (NC1) and cells without transfection (Figure 3A, 3B). Overexpression of S100A9 increased the levels of phospho-AKT1^Thr308^, Vimentin, ICAM-1 and decreased E-cadherin. However, A-674563 prevented the upregulation of phospho-AKT1^Thr308^, Vimentin, ICAM-1, and attenuated the inhibition of E-cadherin expression which was caused by overexpression of S100A9 using LV-S100A9 (Figure 3C). Moreover, HP75 cells were also transfected with S100A9-shRNA-LV or a non-targeting control shRNA lentivirus vector (NC2). Our results demonstrated that shRNA against S100A9 effectively silenced S100A9 expression (Figure 3D, 3E), resulting in the suppression of phospho- AKT1^Thr308^, Vimentin, ICAM-1 as well as promotion of E-cadherin (Figure 3F). After exposure for extra SC79 (AKT1 agonist), Western blot analysis affirmed that the S100A9-shRNA-LV-induced alterations of Vimentin, ICAM-1 and E-cadherin were partially reversed (Figure 3F). These data validated that intracellular S100A9 was also able to activate AKT1 and engender EMT in HP75 cells.

### Intracellular S100A9 stimulates growth of HP75

**Figure 4:**
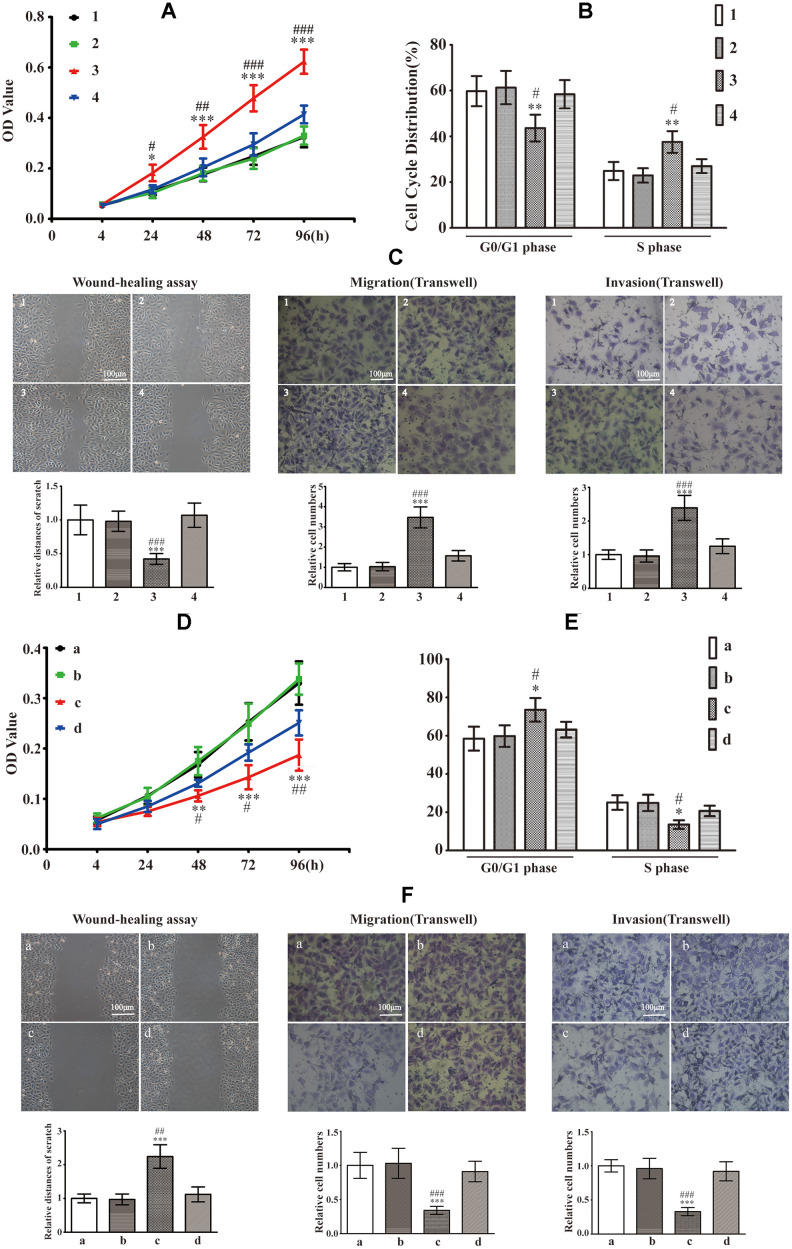
**Effects of intracellular S100A9 on proliferation, migration and invasion in PA.** (**A**) HP75 cell viability in control group (group 1), NC1 group (group 2), LV-S100A9 group (group 3) and LV-S100A9 + A-674563 group (group 4) was tested using CCK-8 after 24, 48, 72 and 96h. (**B**) The cell cycle distribution was determined by FCM in each of the four groups (groups 1, 2, 3, 4) after 96h. (**C**) Wound-healing assay and transwell system were used to measure the migration and invasion of HP75 cells in the four groups (groups 1, 2, 3, 4). (n=5. ^*^P<0.05; ^**^P<0.01; ^***^P<0.001, compared with group 1. ^#^*P*<0.05; ^##^*P*<0.01; ^###^*P*<0.001, compared with group 4). (**D**) CCK-8 to observe the proliferation of HP75 cells in control group (group a), NC2 group (group b), S100A9-shRNA-LV group (group c) and S100A9-shRNA-LV + SC79 group (group d) for 24, 48, 72 and 96h. (**E**) The cell cycles were estimated in group a, b, c, and d using FCM. (**F**) The migration and invasion were also investigated using wound-healing assay and transwell system in groups a, b, c, and d. (n=5. ^*^P<0.05; ^**^P<0.01; ^***^P<0.001 *vs*. group a. ^#^*P*<0.05; ^##^*P*<0.01; ^###^*P*<0.001*vs*. group d).

As depicted in Figure 4A, reconstitution of S100A9 expression augmented cell proliferation at 24, 48, 72 and 96h. The cell cycle distributions found that S100A9-overexpressing cells exhibited a decrease in the G0/G1 phase ratio and an increase in S phase proportion (Figure 4B). Upregulation of S100A9 also promoted cell migration and invasion (Figure 4C). However, A-674563 blocked intracellular S100A9-induced tumor growth (Figure 4A–4C). Furthermore, knock down of S100A9 inhibited HP75 cell proliferation, cell cycle, migration and invasion. However, S100A9-shRNA-LV-mediated repression in HP75 growth was partially restored by SC79 (Figure 4D–4F). It must be emphasized that the cell cycle G2 was not been influenced after alteration of S100A9 levels.

### Upregulation of S100A9 in human IPA

**Figure 5:**
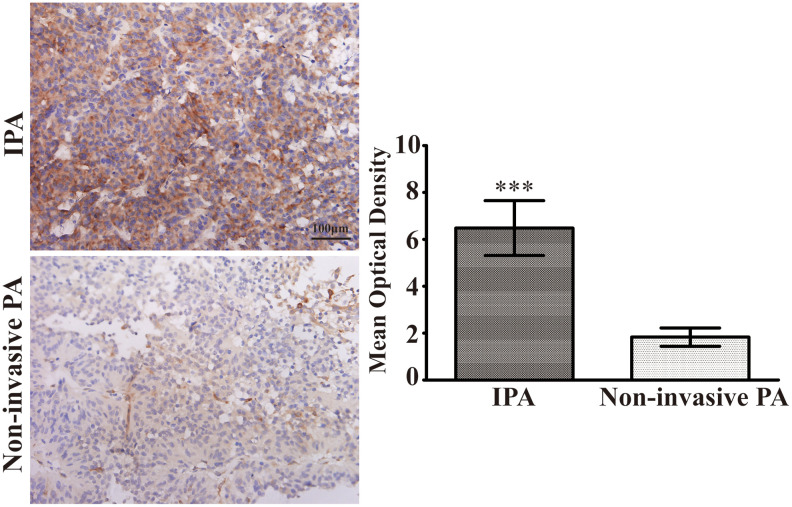
**Upregulation of S100A9 in IPA clinical specimens**. Immunohistochemical staining(×400) of S100A9 in non-invasive PA and IPA. The mean optical density was used for statistical quantification of S100A9 in non-invasive PA and IPA. (^***^P<0.001 *vs*. non-invasive PA).

To assess the clinical relevance between S100A9 expression and PA invasiveness, 80 paraffin-embedded PA clinical tissues (Table 1) were investigated using IHC. These specimens consisted of 40 non-invasive PA (Knosp grade 0 and I) and 40 IPA(Knosp grade II, III and IV). As described in Figure 5, the expression levels of S100A9 were higher in IPA than noninvasive PA. strong immunostaining for S100A9 was observed in 82.5% (33/40) of IPA, while moderate immunostaining for S100A9 was found in other 17.5% (7/40) of IPA samples. By contrast, only 30% (12/40) of noninvasive PA showed high S100A9 expression, and 65% (26/40) of these cases showed low to moderate immunoreactivity for S100A9. However, S100A9 was absent in 30% (12/40) of noninvasive PA. Our data also suggested that there was no significant correlation between the S100A9 levels and age, gender or hormone secretion sub-types (Table 2). These results proved that S100A9 expression was increased in IPA compared with noninvasive PA, and could therefore be a significant biomarker for predicting and judging the invasion characteristics of PA.

**Table 1:** Clinical characteristics of 80 pituitary adenomas.

	**n**	**%**
**Gender**		
Male	43	53.75
Female	37	46.25
**Age**(Years)		
≥45	46	57.50
<45	34	42.50
**Hormone secretion subtype**		
Non-function	41	51.25
Function	39	48.75
PRL	16	20.00
GH	15	18.75
ACTH	8	10.00
**Knosp grade**		
0	11	13.75
I	25	31.25
II	22	27.50
III	12	15.00
IV	10	12.50

**Table 2:** Correlation between clinical features and the expression of S100A9 in 80 pituitary adenomas.

**Characteristics**	**Knosp grade**		***P***
	**0 and I**	**II, III, and IV**	
**Age(years)**	19(≥45)		0.103
	17(<45)		
		27(≥45)	0.083
		17(<45)	
**Gender**	20(Male)		0.319
	16(Female)		
		23(Male)	0.452
		21(Female)	
**Hormone secretion subtype**	15(Non-function)		0.176
	21(Function)		
		26(Non-function)	0.194
		18(Function)	
	9(PRL)		0.097
	7(GH)		
	5(ACTH)		
		7(PRL)	
		8(GH)	0.115
		3(ACTH)	

In order to avoid the different expression of S100A9 in IPA and non-invasive pituitary adenomas, clinical samples were divided into Knosp grade 0 and I, or Knosp grade II, III, IV. The expression of S100A9 in different ages, genders and hormone secretion subtypes were analyzed, respectively.

### Intracellular and extracellular S100A9 improve invasion in primary PA

The primary PA cells P1 and P2 (Clinical information was listed in Table 3) were divided into control, NC1 and LV-S100A9 group. The wound-healing (Figure 6A) and transwell assay (Figure 6B) showed that upregulation of S100A9 enhanced migration and invasion, respectively. Next, the P1 and P2 cells were also divided into control, NC2 and S100A9-shRNA-LV group. Figure 6C, 6D suggested that knock down of S100A9 suppressed migration and invasion after 16h. Finally, 10μg/mL recombinant human S100A9 protein was added into P1 and P2 cells, the abilities of migration and invasion were increased in P1 and P2 cells by the extracellular S100A9 (Figure 6E, 6F).

**Figure 6:**
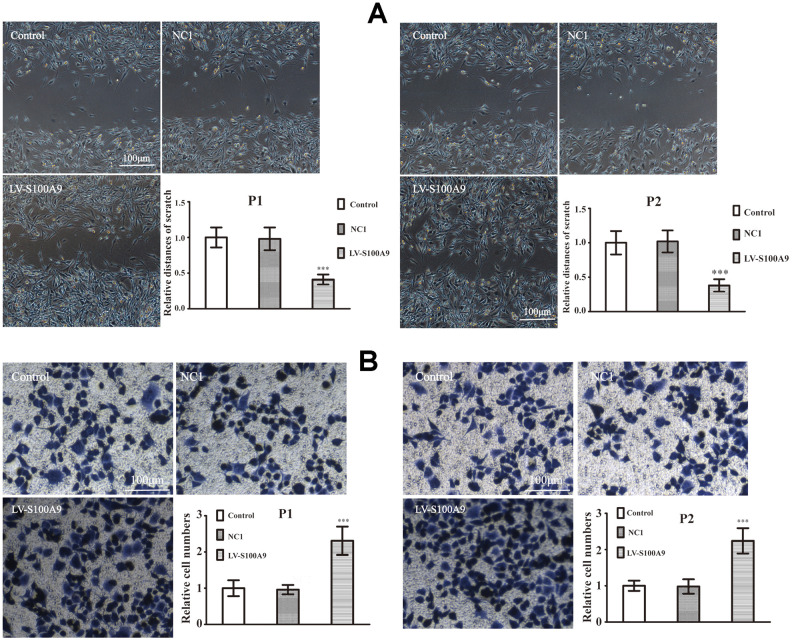
**The effects of S100A9 in primary PA.** (**A**, **B**) Wound-healing assay and transwell system were used to measure the migration and invasion of P1 and P2 cells in control, NC1 and LV-S100A9 groups (n=5. ^***^P<0.001, compared with control).

**Figure 6:**
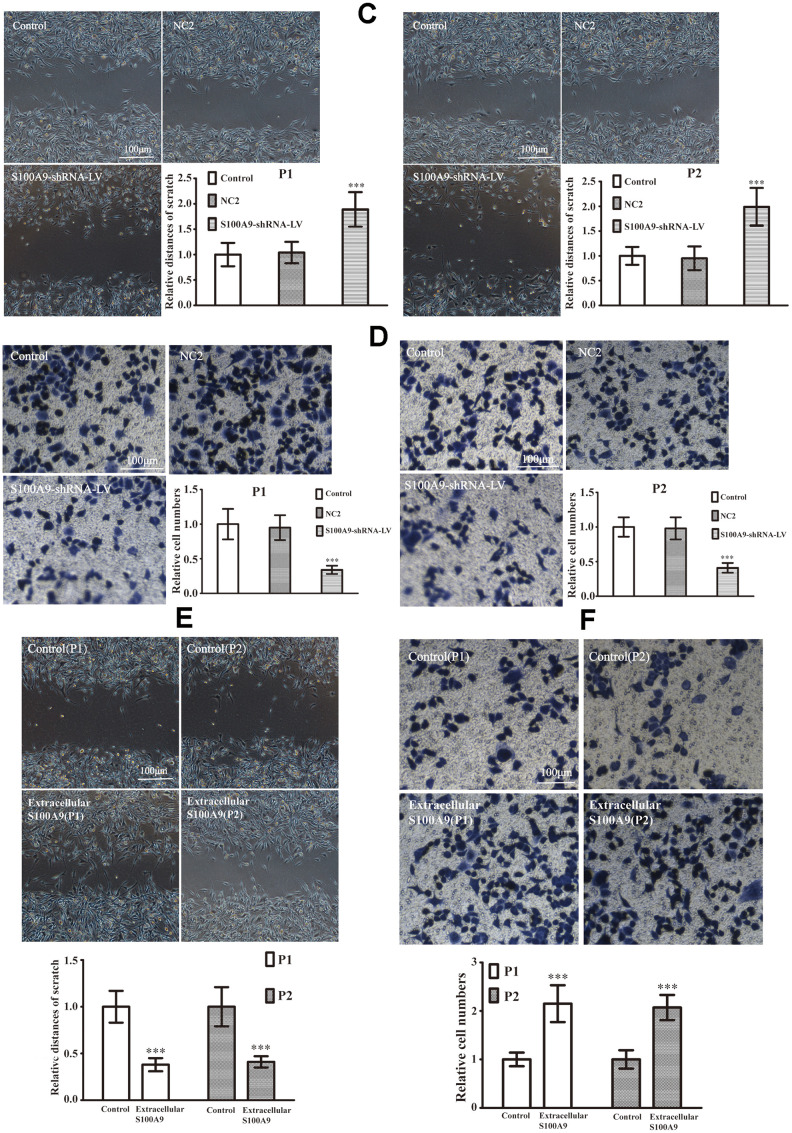
**The effects of S100A9 in primary PA.** (**C**, **D**) The downregulation of S100A9 impeded migration and invasion in primary PA (n=5. ^***^P<0.001, compared with control). (**E**, **F**) The cell migration and invasion were also analyzed after treatment with the recombinant S100A9 protein (n=5. ^***^P<0.001 vs. Control).

**Table 3:** Clinical Characteristics of two pituitary adenomas.

	**Gender**	**Age(years)**	**Hormone secretion subtype**	**Knosp grade**
**Patient 1**	male	52	Non-function	II
**Patient 2**	female	43	PRL	II

## DISCUSSION

S100A9 has been found to be strongly over-expressed in many tumors, leading to carcinogenesis, tumor development, and metastasis [16]. Several lines of evidence have reported that higher levels of S100A9 are associated with shorter survival in patients with tumors [6, 11, 26]. It is notable that S100A9 may serve as a tumor biomarker in serum, urine, and digestive fluid for early molecular diagnosis [27–29]. Although PA is a benign neoplasm, the characteristic of invasive growth is often related to an unfavorable prognosis. In this work, we demonstrated that the levels of S100A9 protein were significantly elevated in IPA compared with noninvasive PA. These results suggested that the expression of S100A9 was positively correlated with invasive phenotype and could be used to estimate IPA.

Previous researches have proposed that S100A9 participated in Ras-mediated tumor progression [30]. Knock down of S100A9 diminished tumor proliferation and invasion through the inhibition of MAPK, NF-kappa B and AKT1 [11, 14]. Nevertheless, S100A9 was not only an inducer but also a downstream target of these signaling pathways [30, 31]. Consequently, S100A9 could be considered as a pivotal target that gathered upstream signals and produced branches of downstream signaling pathways in the tumor development. Additionally, tumor cells were able to secrete S100A9 protein to extracellular stroma, stimulate accumulation of inflammatory cells and offer a pre-metastatic niche in the tumor microenvironment [32–34]. Furthermore, these extracellular S100A9 proteins also could bind to tumor surface receptors (RAGE, TLR4, CD147) and provoke intracellular responses to sustain malignant growth. Consistent with these results, our data confirmed that intracellular and extracellular S100A9 improved PA proliferation, migration and invasion through phosphorylation of AKT1, supporting S100A9 as a novel molecular target for the prediction and therapy of IPA. However, AKT1 maybe not be the sole signal transduction pertaining to S100A9-mediated invasion of PA, and more details need to be elucidated in future studies.

EMT is a biological process during which polarized epithelial cells lose epithelial phenotype and acquire mesenchymal characteristics [35]. Usually, EMT is accompanied with increases in Vimentin and ICAM-1 as well as downregulation of E-cadherin [36, 37]. To date, numerous studies have shown that EMT occurs in various tumors [38], and this pathological transformation has also been found to associate with PA invasion. In this study, our data demonstrated that S100A9 raised Vimentin and ICAM-1, but attenuated E-cadherin, resulting in EMT occurrence, PA growth and invasion. We also confirmed that the formation of EMT by S100A9 was attributed to the activation of AKT1. After incubation with an AKT1 inhibitor, S100A9-induced proliferation, invasion and EMT were neutralized in PA. By contrast, EMT and PA invasion were prevented by the downregulation of S100A9, but these inhibitions could be rescued and restored by an AKT1 agonist.

In summary, the high expression of S100A9 in IPA suggested its possible involvement in IPA pathogenesis. We substantiated that S100A9 activated AKT1, triggering off EMT and IPA progression. Our identification supports the targeting of S100A9 as a novel tool for the detection, diagnosis, prognosis, and molecular therapy of IPA.

## MATERIALS AND METHODS

### Reagents

Dulbecco’s modified Eagle’s medium (DMEM), Minimum Essential Medium (MEM), fetal bovine serum (FBS) and horse serum were purchased from Gibco (San Francisco, California). The lentiviral vectors, polybrene and puromycin were purchased from GeneChem Co., Ltd (Shanghai, China). RNAiso Plus, Primescript RT reagent kit, the primers and DNA polymerase were from TaKaRa Biotechnology (Japan). RIPA lysis buffer, phosphatase inhibitor, SDS-PAGE gels, phenylmethanesulfonyl fluoride (PMSF), cell counting kit-8 (CCK-8), the ECL kit, propidium iodide (PI) and RNase were purchased from Beyotime Institute of Biotechnology (Beijing, China). Recombinant human S100A9 protein was purchased from ABCAM (MA, USA). The fibronectin and Matrigel were bought from Solarbio Science Technology Co., Ltd. (Beijing, China). A-674563 (AKT1 inhibitor) and SC79 (AKT1 agonist) were purchased from MedChem Express (NJ, USA). The primary antibodies: rabbit anti-human S100A9 and rabbit anti-human phospho-AKT1^Thr308^ were purchased from ABCAM (MA, USA); mouse anti-human total-AKT1, mouse anti-human E-cadherin, mouse anti-human Vimentin, mouse anti-human ICAM-1, and mouse anti-human GAPDH were purchased from Santa Cruz Biotechnology (California, USA). Horseradish peroxidase-conjugated secondary antibodies were purchased from Zhongshan Golden Bridge Biotechnology (Beijing, China). Type I Collagenase was bought from Sigma (USA).

### Cell culture

Human pituitary adenoma cell line HP75 was purchased from ATCC and maintained in DMEM containing 2.5% fetal bovine serum, 15% horse serum, and 1% penicillin-streptomycin at 37°C in 5% CO_2_ atmosphere. In addition, the primary PA cells were extracted from two pituitary adenoma patients. Patients’ consent and approval from the Institutional Research Ethics Committee of Chongqing Medical University were obtained for research purposes (No.2020501). Fresh tumor tissues were gathered and washed by PBS in ice. Then the tumor mass was cut into small pieces (1mm^3^). The tissue fragments were digested with Type I Collagenase for 100min at 37 °C. After adding equal amounts of 10% FBS–containing MEM medium, the cell suspension was filtered through 200 Mo filter. Then cell suspension was centrifuged and washed by PBS three times, cell pellet was resuspended in 10% FBS-containing MEM medium and maintained in a 5% CO2-humidified atmosphere at 37 °C. The two primary PA cells were named as P1 and P2, respectively. The clinical information for patients were listed in Table 3.

### S100A9 overexpression and knockdown in PA by Lentiviral particles

The S100A9 lentiviral vector (LV-S100A9) used for the overexpression of S100A9 and matched empty lentivirus (NC1), as well as the lentiviral constructs expressing S100A9 shRNA (S100A9-shRNA-LV, sequence: AGGAGTTCATCATGCTGAT) and corresponding negative control lentivirus (NC2) were purchased from GeneChem Co., Ltd (Shanghai, China). The transfection details were described previously [39]. In brief, one day before transfection, 6 × 10^4^ cells were seeded in 6-well plates. These lentiviral vectors and 5μg/ml polybrene were introduced into HP75 cells. Green fluorescent protein (GFP) was detected using a fluorescence microscope after 72h. Puromycin (2μg/mL) was used to purify the infected cells. Increased or decreased expression of S100A9 was verified by Real-Time PCR and western blot analysis after 96h.

### Real-time PCR

Total RNA from HP75 cells was isolated using RNAiso Plus. After measuring the RNA concentrations using a spectrophotometer, the RNA samples were reverse-transcribed into cDNA using the Primescript RT reagent kit. The S100A9 primer sequences were: forward 5’-TGGCTCCTCGGCTTTGACAGAG T-3’ and reverse 5’-TGGGTGCCCCAGCTTCACAGA-3’. The GAPDH primer sequences were: forward 5’-CTTTGGTATCGTGGAAGGACTC-3’ and reverse 5’-GTAGAGG CAGGGATGATGTTCT-3’. The amplification conditions were as follows: 95°C for 30 s, followed by 40 cycles at 9°C for 5s, and 60°C for 40 s. The relative multiples in mRNA levels were calculated according to the 2^-ΔΔCT^ method [11, 40].

### Western blot

Cells were harvested and lysed using RIPA lysis buffer containing 1%PMSF and 0.1% phosphatase inhibitor. The protein samples (30μg) were separated by SDS–PAGE and transferred onto PVDF membranes. After incubation with primary antibodies overnight at 4°C, including S100A9 (1:200), GAPDH (1:1000), total-AKT1 (1:1000), phospho-AKT1^Thr308^ (1:200), E-cadherin (1:600), Vimentin (1:800), and ICAM-1 (1:600), the PVDF membranes were washed three times with TBST buffer and incubated with a secondary antibody (1:5000) for 50min at 37°C. Next, the membranes were washed three times in TBST buffer, and the amount of protein in each band was determined using ECL reagent and quantified using the Quantity One 4.6 computer software (Bio-Rad, Hercules, California).

### CCK-8 assay

HP75, P1 and P2 cells were treated under various conditions and implanted in 96-well plates at a density of 3000 cells/well. For 24, 48, 72, and 96 h, 10μL CCK-8 and 100μL medium were mixed and added to each well for another 1 h. The absorbance values were measured at 450nm using an enzyme-labeled instrument.

### Flow cytometry analysis

Cells (6 × 10^5^) were collected and fixed in 70% ethanol overnight at 4°C. Then cells were incubated with 10 mg/ml PI and 10 mg/ml RNase at 37°C for 20 min in the dark. The cell cycle distribution was analyzed by flow cytometry (BD Bioscience, Franklin Lakes, New Jersey).

### Transwell chamber

Cell migration and invasion were determined using transwell inserts (8μm pore). The backside of the filters was covered with fibronectin (250μg/50μl). For the invasion test, the filters of the upper compartment were pre-coated with Matrigel (500μg/100μl). 2 × 10^5^ cells (for invasion) and 5 × 10^4^ cells (for migration) were introduced into upper compartment with serum-free medium, and 10% FBS medium was prepared in lower compartment. After 16h, the cells in the upper compartment were removed, and the cells in the bottom compartment were fixed with 4% paraformaldehyde for 20min. The number of cells across the transwell insert was counted following staining with crystal violet.

### Wound-healing assay

Cells (2×10^5^) were implanted in 6-well plates and cultured to reach 80% confluence. Wounds were created by scraping the cells with a sterile 10μl sterile pipette tip. Then, these cells were washed with PBS and cultured under different conditions for 16h. The widths between the edges of the injured monolayer were quantified to assess migrating distances using the T-Scratch software.

### Patients and specimens

Patient permit and approval from the Institutional Research Ethics Committee of Chongqing Medical University were achieved (No.2020501). The clinical information for all of the patients was listed in Table 1. The tissues acquired in this work were from 80 PA patients who underwent surgery between 2013 and 2018 at the Second Affiliated Hospitals of Chongqing Medical University (Chongqing, China). None of the PA patients had received any prior therapy. The pathological diagnosis was reviewed by two independent pathologists. Knosp grades II, III, and IV were defined as IPA. Knosp grades 0 and I were considered to be non-invasive pituitary adenomas.

### Immunohistochemistry (IHC)

IHC was performed as previously described [5]. An S100A9 primary antibody (1:100) was used to survey the S100A9 levels in human PA specimens. Blank controls, treated with PBS instead of the S100A9 primary antibody, showed no positive immunoreactivity. After staining, the slides were observed using a microscope (DM6000 B; Leica, Wetzlar, Germany). The levels of S100A9 were calculated as both the percentage of positive cells and the color intensity. The percent of positive cells was scored as follows: 0 (negative), 1 (less than 10% positive PA cells), 2 (10–50% positive PA cells), and 3 (more than 50% positive PA cells). The immunostaining intensity was classified as follows: 0 (absent), 1 (light yellow), 2 (yellowish brown), and 3 (brown). The staining index (SI)=proportion score × intensity score. Ultimately, SI of 0 was categorized as negative, 1–2 as low, 3–4 as moderate, and 6 or 9 as high.

### Statistical analysis

Statistical analyses were performed using SPSS 19.0. Significant differences among groups were analyzed by the *t*-test, ANOVA and X^2^-square test. All data are presented as the mean ± SD. *P* <0.05 was considered to be statistically significant.
